# Supplementary figures and images for: Distinct functions of cardiac β-adrenergic receptors in the T-tubule vs. outer surface membrane
Source: eLife. 2025 Sep 18;14:e84243. doi: 10.7554/eLife.84243 (PMC12578441; doi:10.7554/eLife.84243)

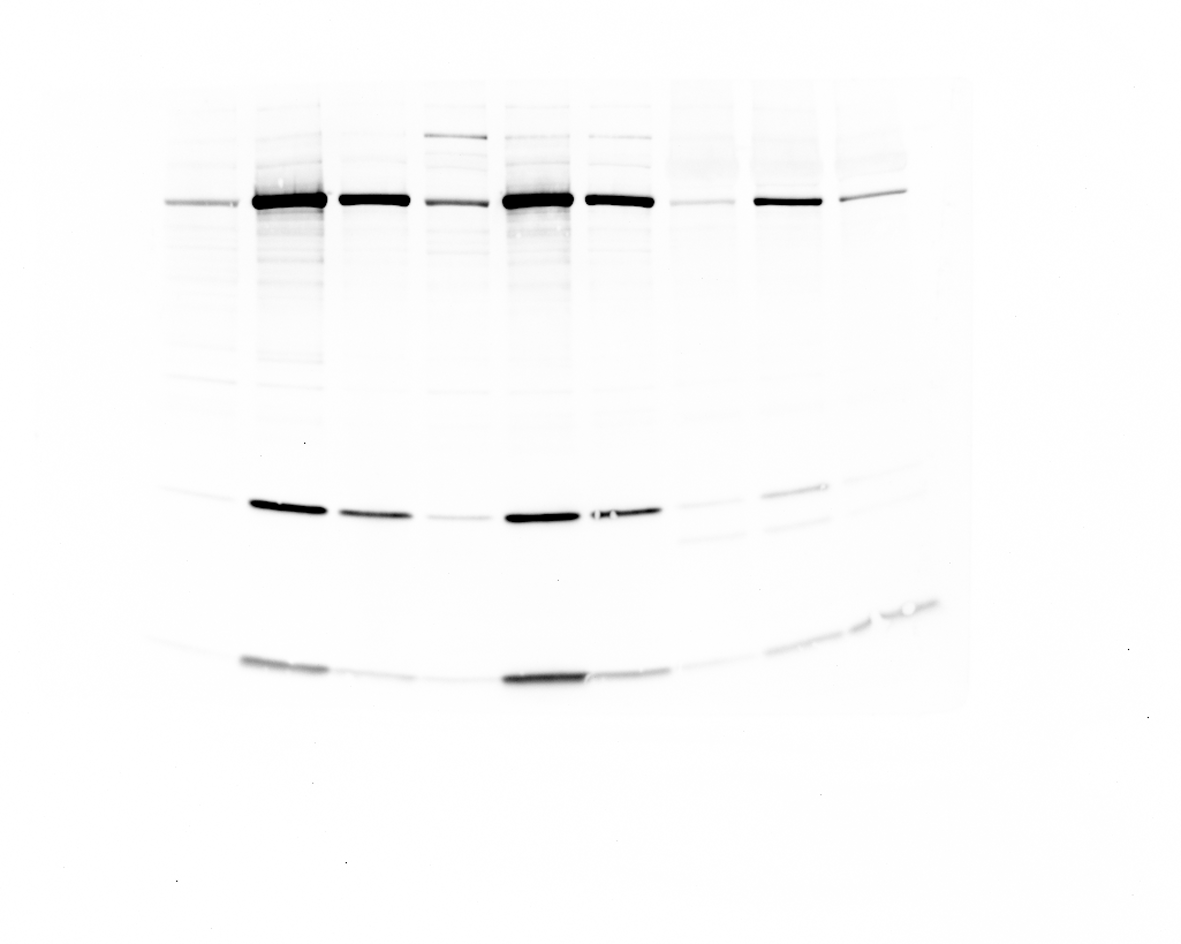

Supplement: Figure 9—figure supplement 1—source data 1. [file elife-84243-fig9-figsupp1-data1.zip › PKA substrates/201218_noyau_4.tif]

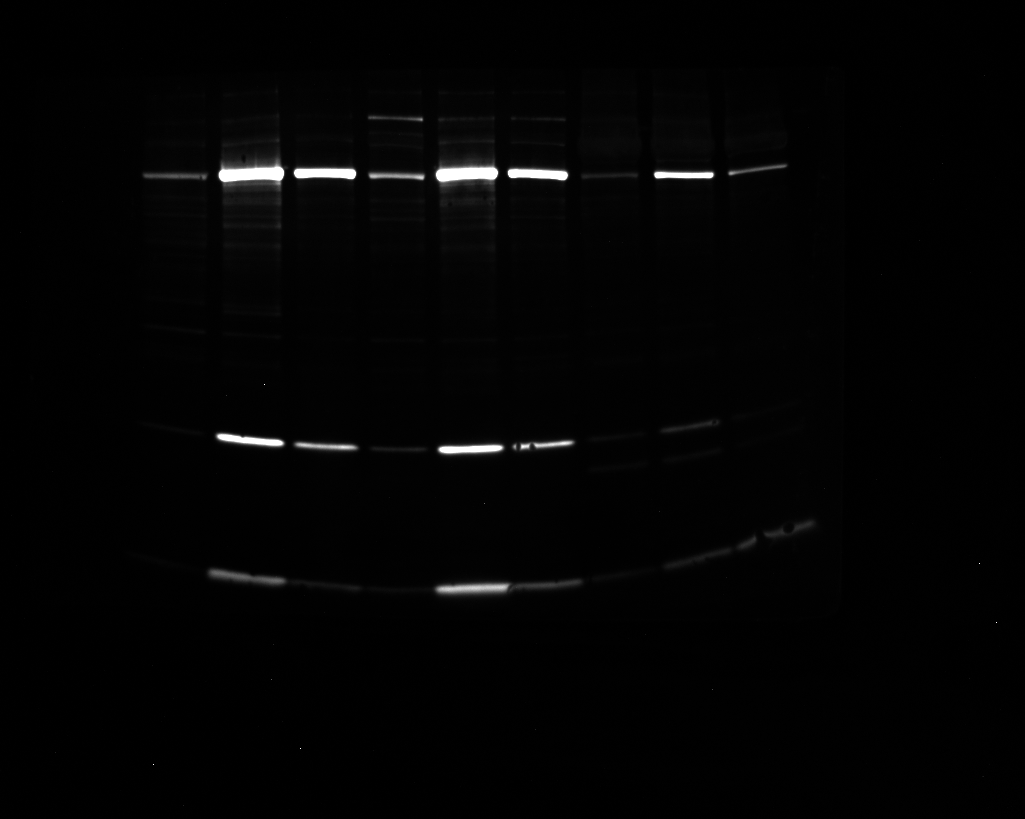

Supplement: Figure 9—figure supplement 1—source data 1. [file elife-84243-fig9-figsupp1-data1.zip › PKA substrates/201218_noyau_4_(Chemi)_raw.tif]

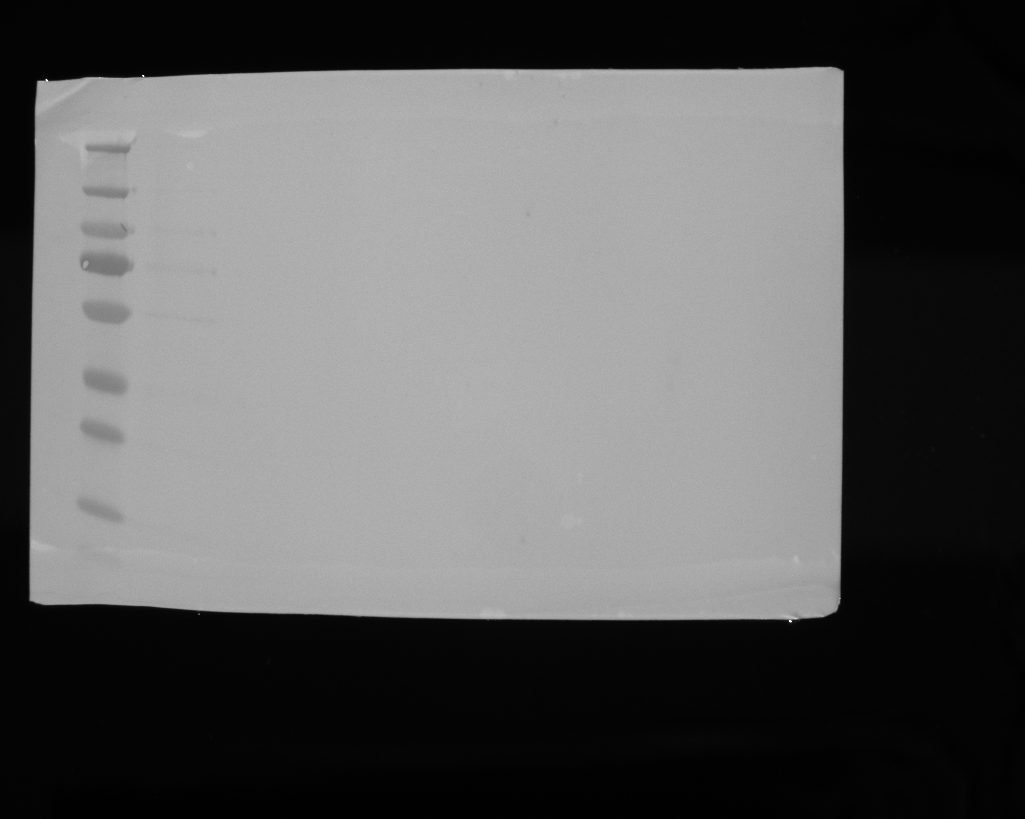

Supplement: Figure 9—figure supplement 1—source data 1. [file elife-84243-fig9-figsupp1-data1.zip › PKA substrates/201218_noyau_4_(Membrane)_raw.tif]

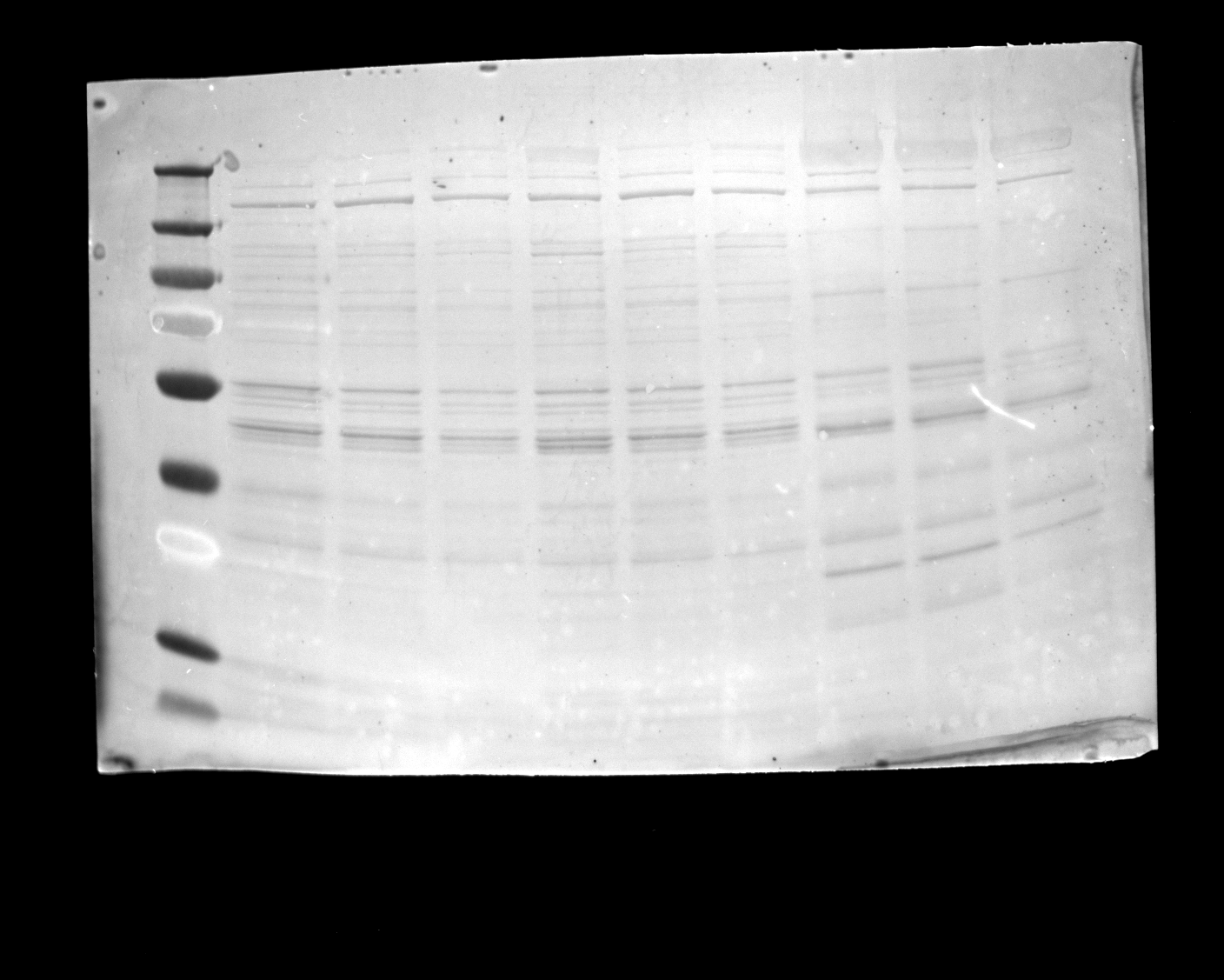

Supplement: Figure 9—figure supplement 1—source data 1. [file elife-84243-fig9-figsupp1-data1.zip › Total prot/201217_noyau_(Protein Gel)_raw_2.tif]

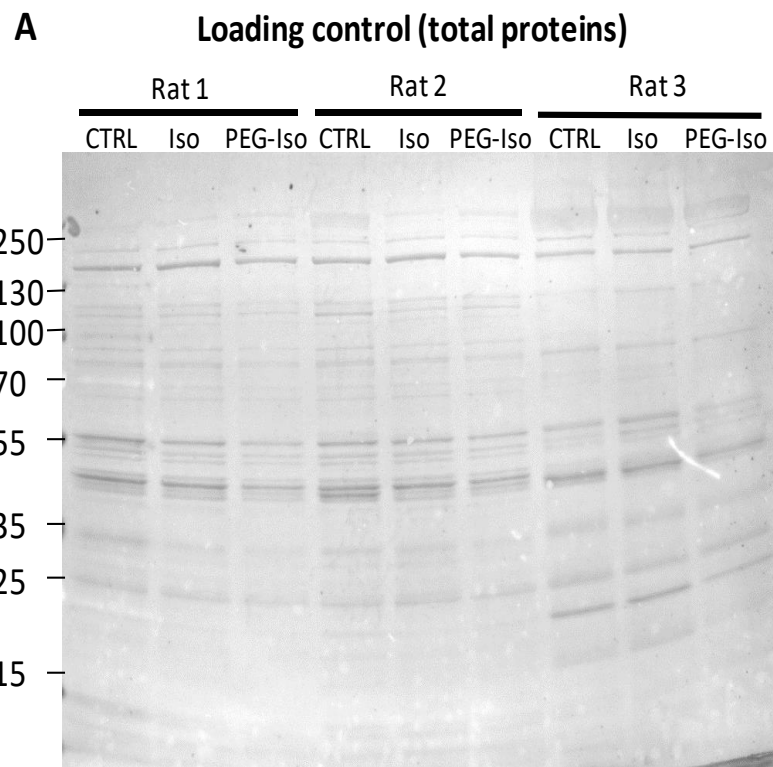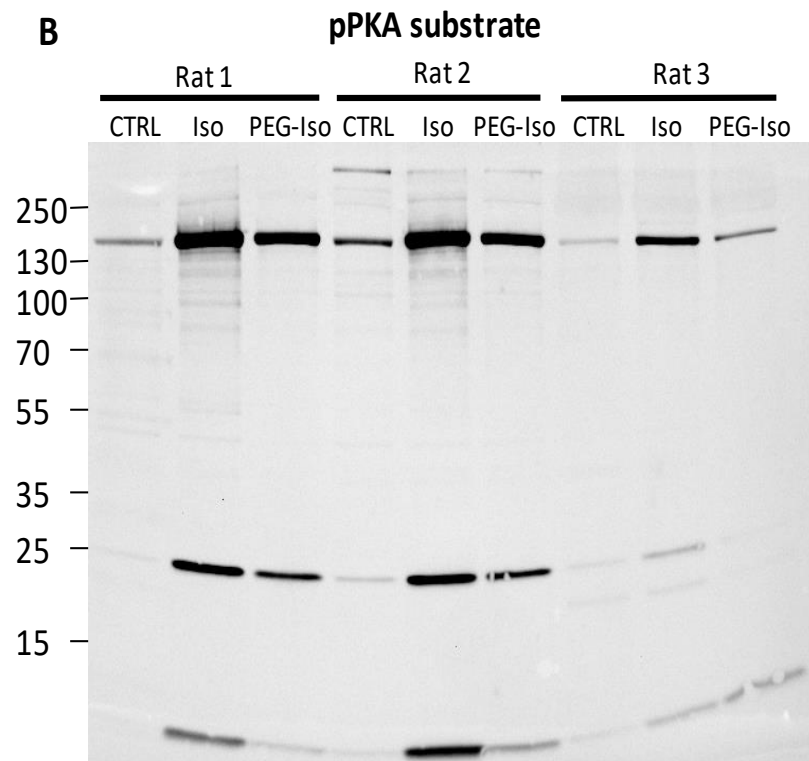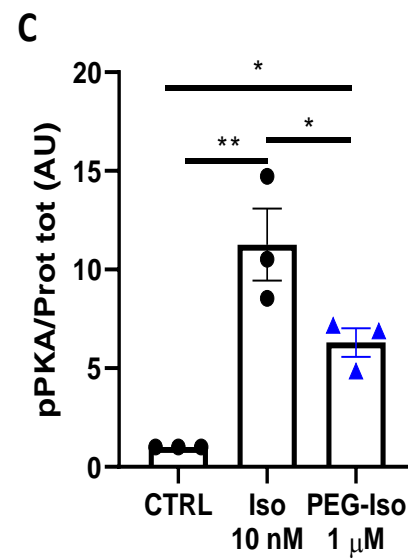

Supplement: Figure 9—figure supplement 1—source data 2. [file elife-84243-fig9-figsupp1-data2.zip › Supplemental Figure 2.pdf]
